# Histopathological Aspects of the Influence of *Babesia microti* on the Placentas of Infected Female Rats

**DOI:** 10.3390/vetsci11010018

**Published:** 2024-01-03

**Authors:** Krzysztof P. Jasik, Anna Kleczka, Aleksandra Franielczyk

**Affiliations:** Department of Pathology, Faculty of Pharmaceutical Sciences in Sosnowiec, Medical University of Silesia in Katowice, Ostrogórska 30, 41-200 Sosnowiec, Poland; olafranielczyk@interia.pl

**Keywords:** *B. microti*, babesiosis vertical transmission, pregnancy, rat placenta

## Abstract

**Simple Summary:**

Babesiosis is a rare zoonotic disease caused by protozoa of the genus *Babesia*. Humans most often become infected with babesiosis as a result of transmission by an infected tick. Blood transfusions, transplanted organs, and vertical transplacental transmission from mother to fetus are much rarer (but possible) modes of transmission. However, the mechanism by which the pathogen enters host cells has not been fully elucidated. This research aimed to show the influence of *B. microti* on the structure of the placenta. Pregnant female rats that were inoculated intraperitoneally with a small inoculation were used for the experiment. Placentas for testing were collected at approximately the 21st day of gestation to give the protozoan a chance to penetrate the placenta and were stained with the Mallory method. Semi-thin sections stained with methylene blue were also analyzed. In addition, the FISH technique was used. The experiment confirmed the presence of inflammation and thromboembolic changes in placentas infected with *B. microti.*

**Abstract:**

Babesiosis is perceived mainly an animal disease; however, awareness that *Babesia* spp. parasites that can cause diseases in humans is increasing significantly. Babesiosis is spread by the bite of an infected tick (*Ixodes* spp.), but it can also be transmitted by transfusion of infected blood and from an infected mother to her child during pregnancy or childbirth. The parasites multiply in the bloodstream and destroy red blood cells. This study aimed to assess the influence of *Babesia microti* on the histological structure of the placenta. Histopathological material collected from pregnant rats infected with *Babesia microti* was used in the experiment. Microscopic images of the placentas were assessed by Mallory staining and by using methylene blue-stained semi-thin sections. In addition, FISH was used to detect parasite DNA. The presence of piroplasms in both maternal and fetal vessels was demonstrated. *Babesia microti* infection caused vacuolization of syncytioblasts and cytotrophoblasts, accumulation of collagen fibers in placental villi, and increased adhesion of erythrocytes to the vascular walls. These results indicate that *Babesia* may influence the course of pregnancy and invite further research on the mechanism of piroplasm penetration into cells.

## 1. Introduction

Babesiosis is a parasitic disease associated with the presence of protozoa of the genus *Babesia* inside the erythrocytes. Pathogens are transmitted by ticks of the genus *Ixodes*, and their reservoir in nature are cattle in the case of *B. divergens* and *B. bovis* and small mammals, mainly rodents, in the case of *B. microti* [1]. Transmission of protozoa to humans can occur as a result of a bite from an infected tick, blood transfusion, organ transplantation, and perinatal infection. In Europe, the percentage of ticks infected with *Babesia* spp. ranges from 0.78% in Switzerland to 51.78% in Austria, while in the United States, human babesiosis has spread with a Lyme-like trajectory over the past two decades [2,3].

The life cycle of *Babesia* spp. involves two different hosts: the Ixodid ticks, in which sexual reproduction takes place, and the vertebrate, in which the parasite reproduces only asexually inside the erythrocyte. The first contact between the parasite and the host’s red blood cells occurs during random events and results first in the reorientation of the apical end of the parasite, then in the formation of a strong connection between the parasite and the host cell. This connection, in turn, leads to invasion of the erythrocyte cell membrane and deeper engulfing of the merozoite inside the vacuole it has created. The parasitic vacuoles disintegrate soon after invasion, and the parasitic membrane remains in direct contact with the erythrocyte cytoplasm for the rest of the intraerythrocytic life stage. After the parasite inside the erythrocyte is ingested by the tick *Ixodidae* spp. while the tick feeds on blood, the protozoans have to cross many cell barriers as they migrate through the body cavities and tissues of the tick, while at the same time undergoing several metamorphic changes that are still only partially understood [4,5]. Gametocytes, which are believed to be already present in the blood absorbed by the tick, mature into dimorphic elongated gametes—ray bodies, which fuse to form diploid zygotes. Zygotes adhere to intestinal epithelial cells, penetrate them, and finally transform into motile kinets that are released into the haemocoel and migrate to various tissues, including alveolar secretory cells of the salivary glands. Here, the parasites reproduce by sporogony, forming colonies of sporozoites [6,7,8].

The incubation period of babesiosis after a tick bite is usually one to six weeks, but parasitemia reaches its peak in one to nine weeks if transmission of the protozoan occurred by blood transfusion. The incubation period depends on various factors, including the recipient’s immune status. *B. microti* infection results in a wide range of clinical manifestations, including asymptomatic infection and non-specific, flu-like symptoms. Serious complications of babesiosis include acute respiratory distress syndrome, disseminated intravascular coagulation (DIC), congestive heart failure, and renal failure [9]. The possibility of transplacental transmission of the pathogen has been suggested in several publications describing cases in which newborns were not transfused and mothers denied that their children had been exposed to a tick bite. Congenital babesiosis has also been encountered as a result of antenatal infection involving the placenta. Reported cases of congenital babesiosis are characterized by asymptomatic maternal infection and development of fever, hemolytic anemia, and infant thrombocytopenia detected between 19 and 41 days postnatally [10,11,12].

Babesiosis in pregnancy may be the cause HELLP (Hemolytic anemia, Elevated Liver enzymes, Low Platelet count) syndrome. For that reason, babesiosis may be the cause is a serious complication of pregnancy that can cause increased maternal and neonatal mortality. It is suggested that for acute symptoms, a differential diagnosis be considered based on geographic area, travel history with possible exposure, and the presence of signs and symptoms consistent with infection [13,14]. Differential diagnosis should include a peripheral blood smear to assess hemolysis and the presence of parasites inside the erythrocytes. To date, no effective diagnostic method has been developed to distinguish between HELLP syndrome and babesiosis, the symptoms of which are very similar [15]. 

Maternal risk factors for severe babesiosis during pregnancy are not well understood. Regardless, it seems that treatment and the mother’s immune response should protect the fetus. Distinguishing babesiosis from other infectious and non-infectious clinical pathologies of pregnancy is essential to ensuring early treatment and homeostatic optimization in mothers and fetuses. Congenital babesiosis is a poorly understood, recently discovered infectious disease that should be considered in the differential diagnosis of febrile, anemic, thrombocytopenic, or neutropenic neonates [16,17,18]. 

Babesiosis and malaria can be successfully treated with a similar combination of antibiotics, and empirical treatment should be initiated as soon as possible to avoid the serious complications of untreated disease. Azithromycin and atovaquone are used to prevent vertical transmission. Patients with babesiosis can also be treated with clindamycin and quinine; however, such treatment is completely inadvisable for pregnant women due to the adverse effects of quinine [19,20].

The main aim of this study is to broaden our knowledge of the histopathological aspects of the impact of *B. microti* on the placenta, which may suggest an adverse effect on fetal development. Understanding the mechanisms of the parasite’s penetration through the placenta should increase knowledge about the need for screening tests in pregnant women to eliminate infection and prevent damage by *B. microti* to the structure of the placenta and the consequent possibilities of complications during pregnancy and fetal malformations.

## 2. Materials and Methods

### 2.1. Tissue Samples

The materials used for the research were the placentas of pregnant female Wistar rats, which were collected on the 20th day of pregnancy (the total duration of pregnancy is about 22–23 days). Ten females in the test group were injected intraperitoneally with an inoculum of *Babesia microti* (reference strain: ATCC^®^ 30221™) three weeks before mating. At that time, three females were injected intraperitoneally with 0.5 mL of sterile saline (control group). The females were then mated using the harem method. 

During the experiment, the state of parasitemia of the infected females was monitored by analyzing blood smears taken from the tail vein. Parasitemia control was performed three times, on the 7th, 14th and 20th days of pregnancy. On day 20 of gestation, the females were euthanized by an isoflurane overdose and the placentas were removed. At the same time, fetal blood smears were checked for infection. 

The study, from infection to killing the animals and harvesting the organs for research, were carried out in accordance with established animal-testing procedures after the researchers obtained the consent of the Local Ethical Committee for Animal Experiments in Katowice—Resolution No. 32/2011 of 23 May 2011. The collection of the material was performed at the Experimental Medicine Center of the Medical University of Silesia in Katowice. Other tests were performed at the Department of Pathology of the Faculty of Pharmaceutical Sciences of the Medical University of Silesia in Sosnowiec.

### 2.2. FISH (Fluorescence In Situ Hybridization) Technique

The autopsy material intended for FISH analysis was fixed in a paraformaldehyde solution and then frozen at −20 °C. Sections with a thickness of approx. 15 µm were obtained using a cryostat. After the sections were rinsed with PBS solution, the genetic material of *B. microti* was detected using a commercial Histology FISH Accessory Kit (DAKO, Carpinteria, CA, USA), according to the manufacturer’s instructions. 

The FISH analysis was conducted with a probe labeled with fluorescein and complementary to the 18S rDNA fragment with the following sequence: 5′-fluoresceins—GCCACGCGAAAACGCGCCTCGA-fluorescein-3′. The oligonucleotides were synthesized at the Institute of Biochemistry and Biophysics of the Polish Academy of Sciences.

After the probe was applied, the material was denatured for 5 min at 84 °C and then hybridized for 30 min at 42 °C. Unbound probes were removed from the preparation with phosphate buffer. The material prepared in this way was analyzed in the Olympus BX60 light microscope at 2200 times magnification. A xenon lamp was used to induce fluorescence. The image was analyzed using a blue filter for fluorescein [21].

### 2.3. Histopathology Examination 

The fragments of the collected placentas were fixed for 1–2 days in Bouin’s solution to obtain slides for routine histological analysis. The fixed material was rinsed with 80% ethanol for 7 days with twice-daily alcohol changes to remove picric acid from the tissues. Then, the material was dehydrated in an alcohol series of increasing concentration. Fixed and dehydrated tissues were overexposed in benzene and embedded gradually in a mixture of benzene and paraffin *v*/*v* 1:1, then in paraffin with a lower melting point, and finally in paraffin with a higher melting point.

The paraffin-embedded material was sectioned using a sled microtome. Paraffin serial sections with a thickness of about 6 µm were obtained. The sections were placed on glass slides that had previously been degreased with alcohol and coated with a mixture of glycerin and thymol-stabilized protein to increase the adhesion of the sections to the slides. The material was applied to prepared slides, covered with a layer of water, and placed on a heating plate (40 °C) to straighten the sections.

Tissue sections were then deparaffinized with xylene and rehydrated with decreasing concentrations of alcohol. Mallory staining was performed using 0.1% acid fuchsin aqueous solution, 1% phosphomolybdic acid, and Mallory’s reagent (0.5 g aniline blue, 2 g orange G, 2 g oxalic acid, and 100 mL distilled H_2_O) [22]. 

### 2.4. Semi-Thin Sections 

Some fragments of placentas were fixed in a buffered, 2.5% paraformaldehyde solution. After it was rinsed in a phosphate buffer, the material was additionally fixed in buffered osmium (VIII) oxide–OsO_4_ for 1 h. The tissues were washed twice (2 × 30 min) with phosphate buffer and dehydrated in the alcohol-acetone series. Finally, the material was embedded in epoxy resin. After a polymerization lasting 3 days at 60 °C, semi-thin sections (0.5 µm thick) were obtained using a Reichert-Jung Ultracut ultramicrotome and stained with methylene blue solution.

Both paraffin sections and semi-thin sections were analyzed using an Olympus BX60 light microscope equipped with an 

DP74 digital camera. Microphotographic documentation was obtained using the Olympus cellSens Standard software version 3 [23].

## 3. Results

### 3.1. FISH

**Figure 1:**
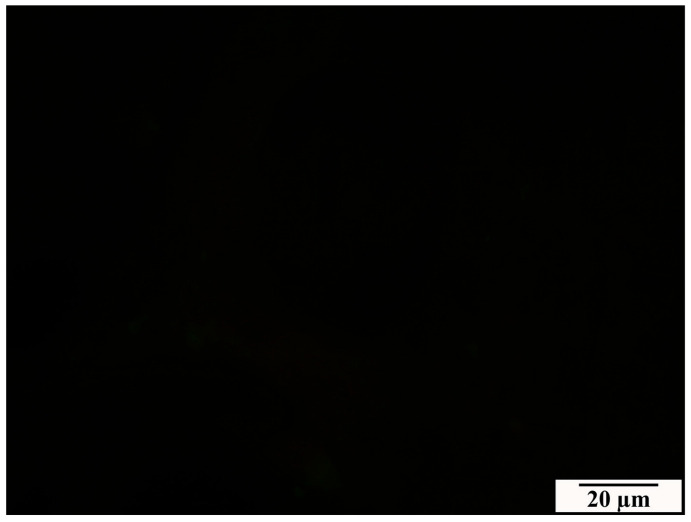
No fluorescence signals in placental sections of control rats.

**Figure 2:**
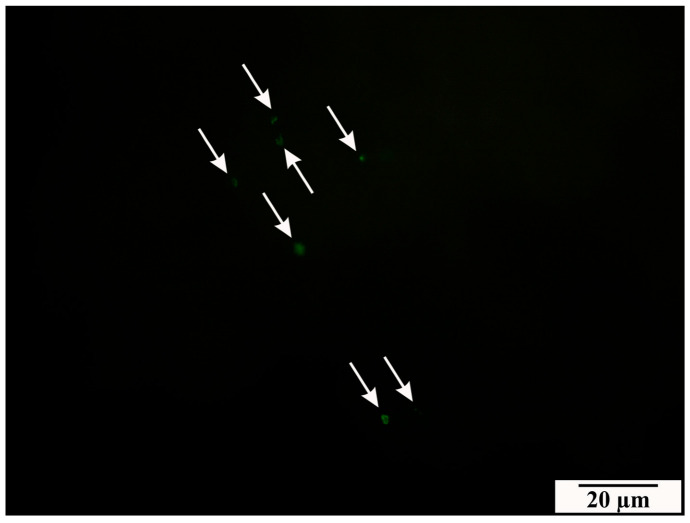
Confirmation of the presence of the pathogen in the placenta by FISH reaction in a cryosection from a rat placenta from the infected group. FISH reaction signals (white arrows) indicate the presence of *B. microti* DNA.

The observations confirmed the presence of *B. microti* in the placental tissues of infected rats. Green spot signals of fluorescence were detected in tissue samples from the infected group when the designed probes annealed to the complementary *B. microti* DNA. Fluorescence signals were present in many fields of observation. No fluorescence signals were detected in the material taken from rats from the control group. FISH results are shown in Figure 1 and Figure 2.

### 3.2. Mallory Staining

#### 3.2.1. Control Group

**Figure 3:**
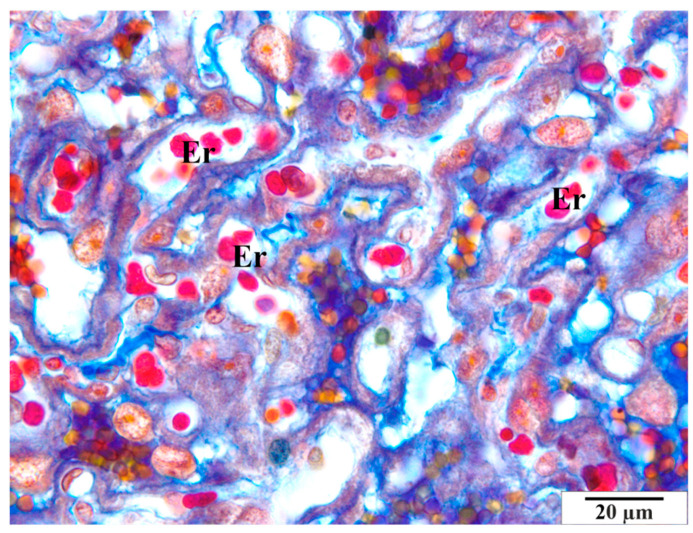
Rat placenta from the control group—placental labyrinth. Image of normal blood vessels with regularly stained erythrocytes of the correct shape (Er). Preparation dyed with met. Mallory (magnification 2200×).

**Figure 4:**
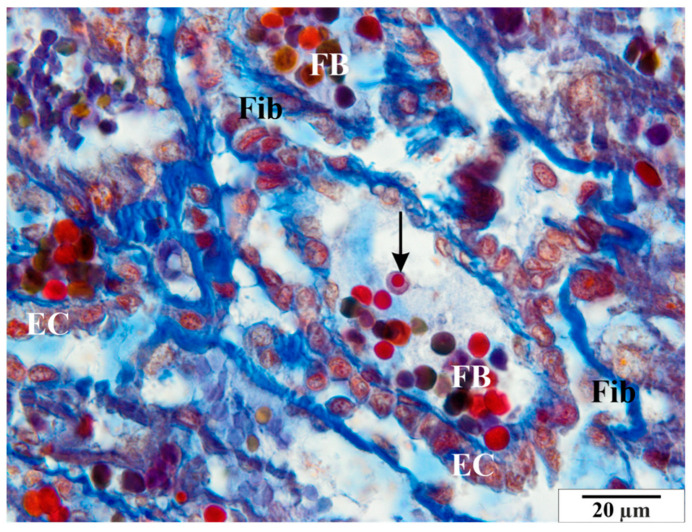
Rat placenta from the control group—placental labyrinth. Image of normal fetal blood vessels. Nucleated erythrocyte (arrow). EC –epithelial cells of villi, Fib—fibrin, FB—fetal blood. Preparation dyed with met. Mallory (magnification 2200×).

**Figure 5:**
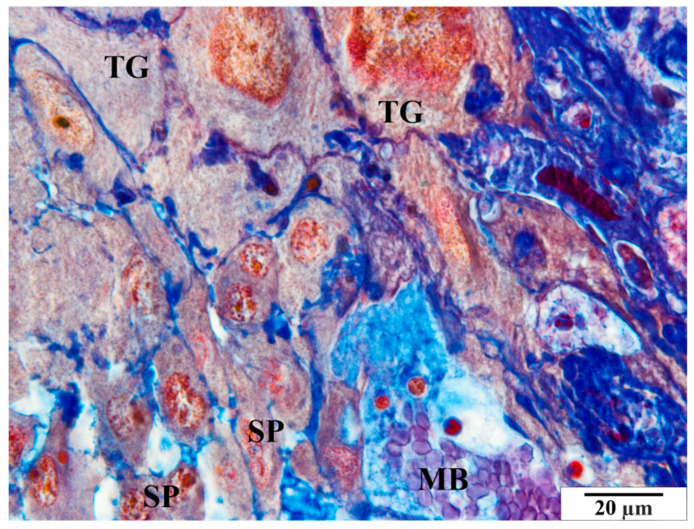
Rat placenta from the control group—basal zone. MB—maternal blood, SP—spongiotrophoblast, TG—giant cells. Preparation dyed with met. Mallory (magnification 2200×).

In the group of rats that were not infected with *B. microti*, no changes in the histological structure of the placentas were observed. Blood vessels, placental villi, and labyrinthine zones remained normal. Histological images of a representative physiological placenta from a control rat are presented in Figure 3, Figure 4 and Figure 5.

#### 3.2.2. Infected Group

**Figure 6:**
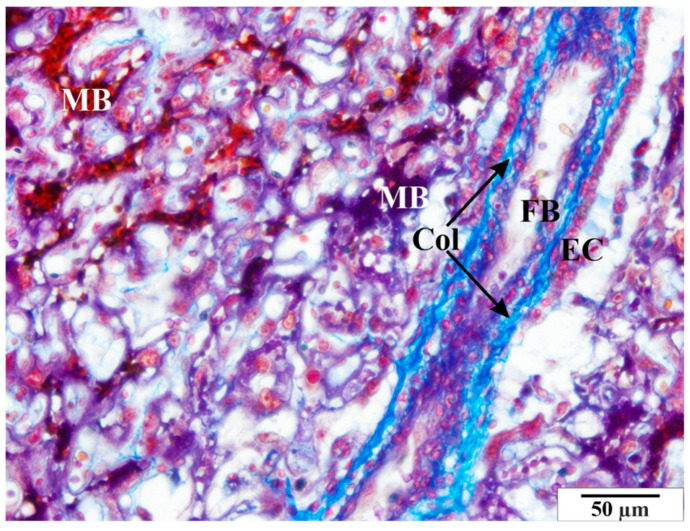
Rat placenta from the infected group—placental labyrinth. Col—collagen, EC—epithelial cells of villi, FB—fetal blood, MB—maternal blood. Preparation dyed with met. Mallory (magnification 2200×).

**Figure 7:**
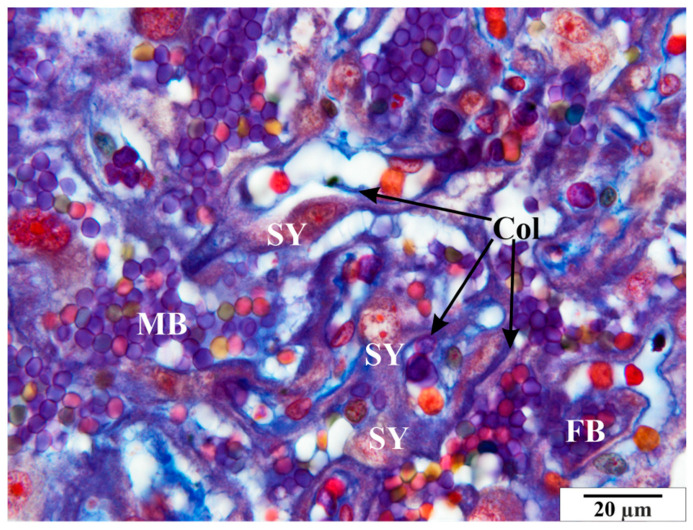
Rat placenta from the infected group—placental labyrinth. Col—collagen, FB—fetal blood, MB—maternal blood, SY—syncytiotrophoblast. Preparation dyed with met. Mallory (magnification 2200×).

Numerous structural changes were observed in the placentas of infected rats, along with abnormalities in red blood cell morphology, such as poikilocytosis, anisocytosis, hyperpigmentation, and hypochromia (Figure 6 and Figure 7). These features were observed in both maternal and fetal vessels. An increased amount of collagen fiber and a widened space were also observed around the blood vessels (Figure 6 and Figure 7).

**Figure 8:**
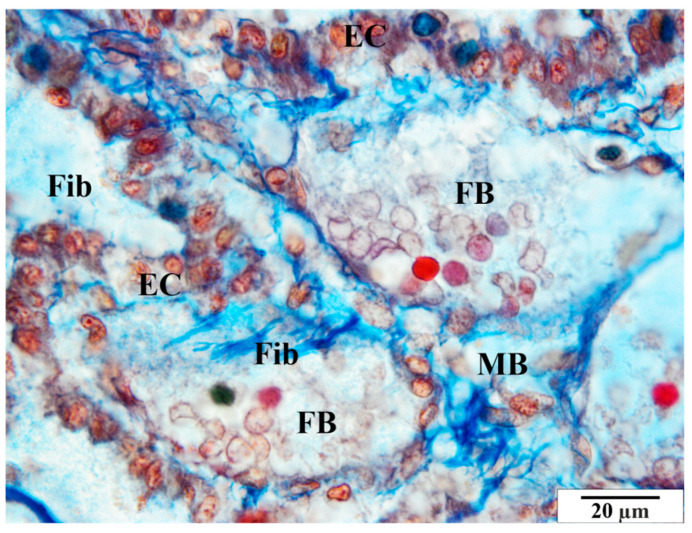
Rat placenta from the infected group—placental labyrinth. EC—villous epithelial cells, Fib—fibrin, FB—fetal vessels, MB—maternal vessel. Preparation dyed with met. Mallory (magnification 2200×).

**Figure 9:**
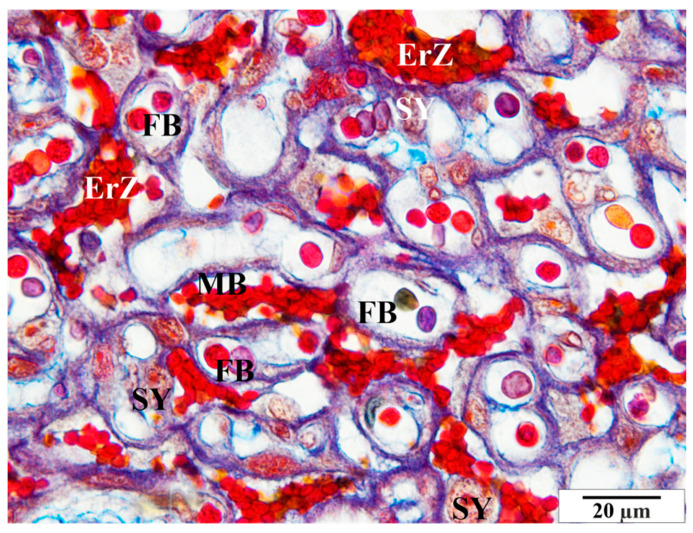
Rat placenta from the infected group—placental labyrinth. ErZ—blood clots, FB—fetal blood, MB—maternal blood, SY—syncytiotrophoblast. Preparation dyed with met. Mallory (magnification 2200×).

**Figure 10:**
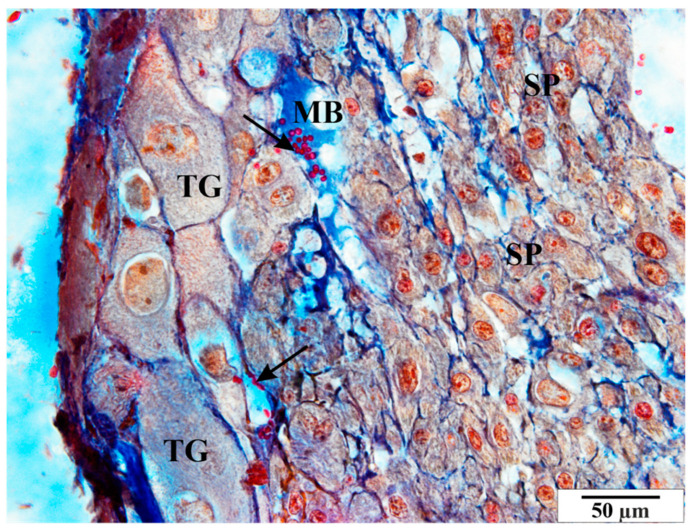
Rat placenta from the infected group—basal zone MB—maternal blood, arrows—erythrocytes adjacent to the vascular endothelium, SP—spongiotrophoblast, TG—giant cells. Preparation dyed with met. Mallory (magnification 2200×).

Other pathological features include the presence of numerous vacuoles within the cells (Figure 6, Figure 7 and Figure 8) and broad bands of fibrin that are much larger than those in the control animals (Figure 8). The microscopic image of the examined placentas also indicated strong hyperemia of this organ. In some cross-sections through the vessels, clear clots were visible (Figure 8 and Figure 9). In addition, the following were observed the characteristic adhesion of blood cells (erythrocytes) to the vascular endothelial cells (Figure 10). 

**Figure 11:**
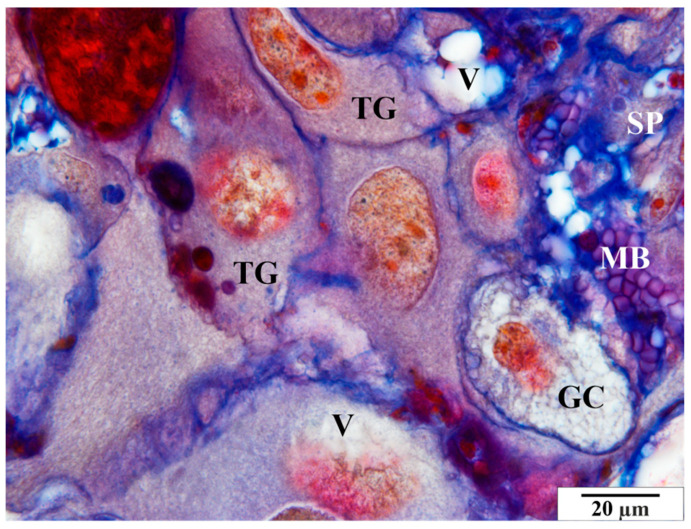
Rat placenta from the infected group—basal zone. GC—glycogen cell, MB—maternal blood, SP—spongiotrophoblast, TG—giant cells, V—vacuoles. Preparation dyed with met. Mallory (magnification 2200×).

In the glycogen cells, as in the animals from the control group, the cytoplasm was highly diffused, frothy, and devoid of storage materials (Figure 11).

### 3.3. Microscopic Image of Semi-Thin Sections Stained with Methylene Blue

#### 3.3.1. Control Group

**Figure 12:**
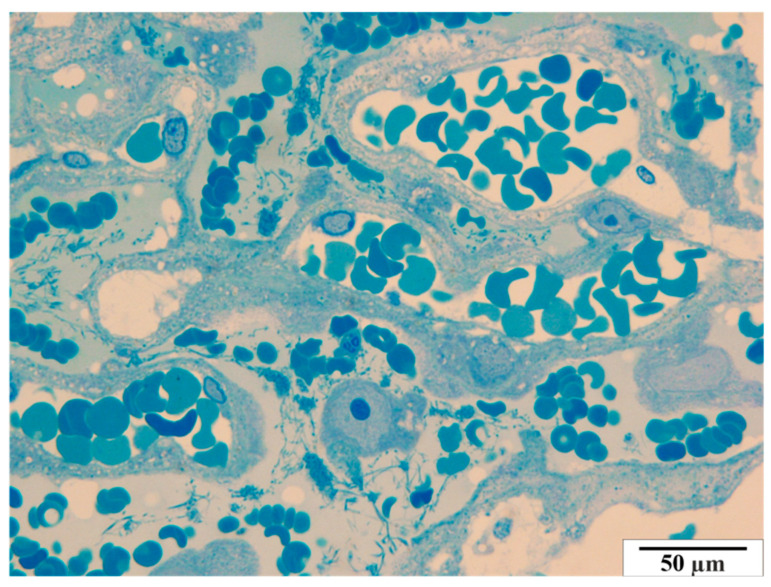
Rat placenta from the control group—placental labyrinth. Semi-thin preparation, stained with methylene blue (magnification 2200×).

**Figure 13:**
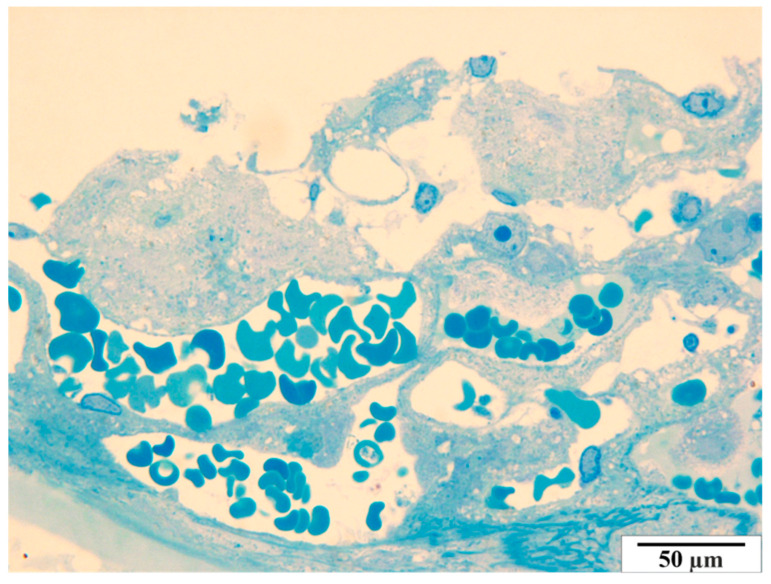
Rat placenta from the control group—placental labyrinth. Semi-thin preparation, stained with methylene blue (magnification 2200×).

In the group of rats that were not infected with *B. microti*, no changes in the histological structure of the placentas were observed. The semi-thin images did not reveal any vascular anomalies. A representative image of a placenta of a rat from the control group in semi-thin preparations is presented in Figure 12 and Figure 13.

#### 3.3.2. Study Group

**Figure 14:**
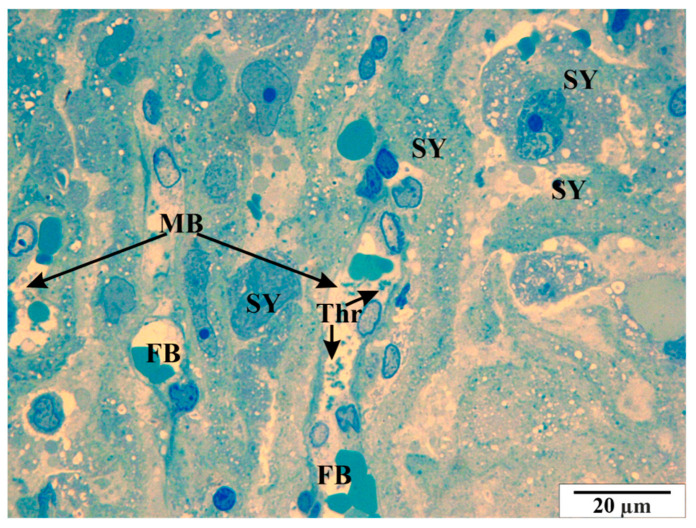
Rat placenta from the infected group—placental labyrinth. Thr—thrombocytes, FB—fetal vessels, MB—maternal vessel, SY—syncytiotrophoblast cells. Semi-thin preparation, stained with methylene blue (magnification 2200×).

**Figure 15:**
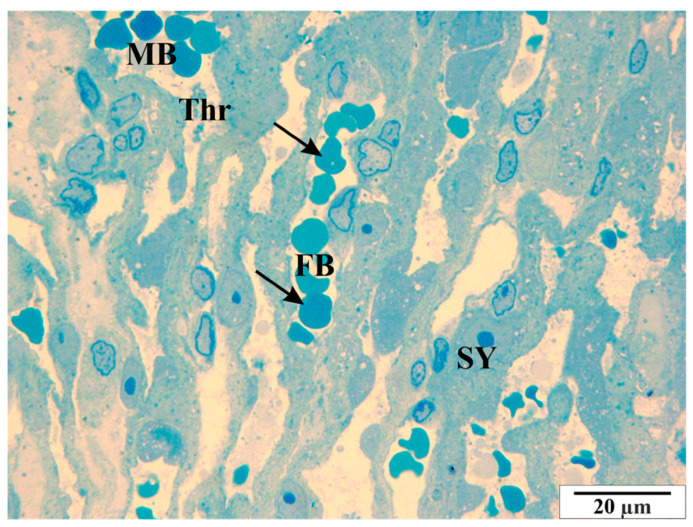
Rat placenta from the infected group—placental labyrinth. Thr—thrombocytes, FB—fetal vessels, MB—maternal vessel, SY—syncytiotrophoblast cells, arrows—*B. microti* erythrocytes. Semi-thin preparation, stained with methylene blue (magnification 2200×).

**Figure 16:**
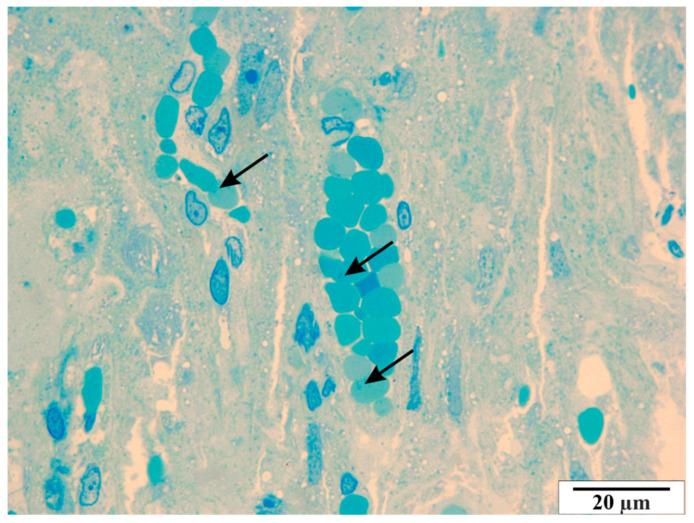
Rat placenta from the infected group. Arrows—*B. microti* infected erythrocytes. Semi-thin preparation, stained with methylene blue (magnification 2200×).

**Figure 17:**
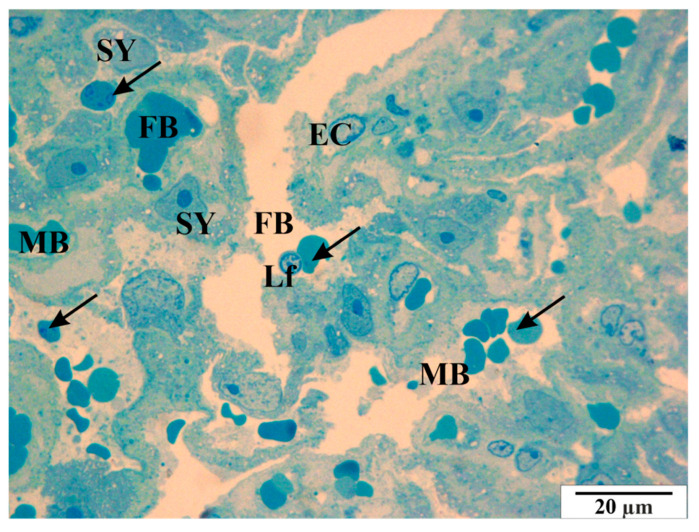
Rat placenta from the infected group—placental labyrinth. arrows—*B. microti* in erythrocytes, EC—villi epithelial cells, FB—fetal vessels, Lf—lymphocyte, MB—maternal vessel, SY—syncytiotrophoblast cells. Semi-thin preparation, stained with methylene blue (magnification 2200×).

Analysis of semi-thin slides revealed thrombocyte conglomerates, both within the vascular lumen and near the endothelial cells (Figure 14 and Figure 15). In some cross-sections of the vessels, erythrocytes are present in a very large number, showing the symptom of congestion (Figure 16). In both maternal and fetal vessels, erythrocytes containing *B. microti* trophozoites are visible (Figure 15, Figure 16 and Figure 17). Numerous small vacuoles are visible in the cytoplasm of both the trophoblast and syncytiotrophoblast cells (Figure 14, Figure 15, Figure 16 and Figure 17).

## 4. Discussion

The placentas of humans and non-human mammals can have differences in the structures that enable the exchange of nutrient metabolites, oxygen, and carbon dioxide between the maternal and fetal blood. In theory, the placenta, as it prevents the blood of the fetus and mother from mixing, protects the developing offspring through appropriate barriers, ensuring, among other things, sterile conditions. Nevertheless, many pathogens cross these barriers, causing congenital diseases. In humans, as in rats and mice, there is a hemochorial placenta, which means that the villi are in direct contact with the mother’s blood. However, the blood placentas of humans and the aforementioned rodents differ in the number of layers of trophoblast cells. A hemomonochorial placenta is typical for humans, while a hemotrichorial placenta is found in rats [23,24]. This variation implies a variation in the number of barriers to be overcome by potential pathogens of multiple mammalian species. Although there are more barriers to overcome in rats than in humans, transplacental infection of rat fetuses has been confirmed.

The formation of the placenta is a dynamic process involving many developmental processes, differentiation of trophoblast cells, and the development of cells that build fetal membranes. This development is determined by genetics, the modulating influence of the maternal environment, and the viability of the fetus [25]. As such, it exhibits the plasticity necessary for the formation of a durable and physiologically efficient placenta.

A properly developed maternal-fetal network is able to adapt to the stresses and challenges of pregnancy. Errors in the regulatory processes related to the control of placental development and function may adversely affect maternal health and fetal development and may also have a lasting impact on postpartum performance [26].

As mentioned above, there are some differences in the structure and function of the rodent placenta compared to the human placenta. There are also clear differences in the biology of pregnancy in rodents and humans. However, there are also significant similarities between species. Placental development in rats and humans shows striking similarities, particularly concerning remodeling of the spiral arteries of the uterus by trophoblasts. Both species show deep trophoblast invasion [24].

The labyrinthine zone present in the fetal part of the rat placenta is located on a flat, wide chorionic plate and constitutes a thick vascular layer that terminates the umbilical cord. It consists of maternal sinusoidal vessels, septal trophoblasts, and fetal capillaries. The outer cytotrophoblast. Beneath the cytotrophoblast are two layers of syncytiotrophoblast (syncytiotrophoblast I and syncytiotrophoblast II). The continuous layers of syncytiotrophoblasts constitute the essential placental barrier [27,28]. The labyrinthine zone develops as pregnancy progresses and accounts for the greater part of the placental volume. 

The human placenta consists of a fetal part (chorion plate) and a maternal part (basal plate), each with well-defined characteristics and functions. The chorionic plate is covered by the amnion. The umbilical cord is usually located in a slightly eccentric position in the placenta, but there are other types of placement, such as marginal umbilical cord placement, wherein the umbilical cord is attached laterally, or membranous attachment of the umbilical cord, wherein the umbilical vessels are present on the surface of the amniotic membranes at some distance from the edge of the placenta, surrounded only by a fold in the amnion, so they are outside the placenta [29,30]. In such a case, if the amniotic membranes rupture, that rupture will lead to the interruption in the continuity of the umbilical vessels, the consequence of which may be bleeding and fetal death. In the placental membranes, the fetal vessels are unprotected. In umbilical vessels, protection is provided by Wharton’s gelatinous connective tissue, which is composed mainly of mucopolysaccharides (hyaluronic acid and chondroitin sulfate) and fibroblasts [31]. The chorionic plate contains vessels that are branches of the umbilical vessels. Originating from two umbilical arteries, chorionic arteries are characterized by centrifugal distribution in their terminal branches, which allows them to supply blood to the villi. The chorionic veins are the result of the fusion of the villous veins (stalk villi, attached to the plate) and usually intersect beneath the chorionic arteries. Chorionic veins give rise to a single umbilical vein [29,32].

The placental villi, which in humans are arranged in a treetop-like pattern, are functionally analogous to the labyrinth zone in rats. Placentas of humans and rats, despite the differences in the thickness of the trophoblast layers, show fundamental similarities and are both classified as hematochorionic placentas. The fetal-maternal interface in humans consists of a single layer of syncytiotrophoblasts and a single layer of cytotrophoblasts, so it is classified as hemomonochorial. In rats, this interface consists of three layers and is referred to as hemotrichorial [33]. 

This difference may affect fetal-maternal exchange across the placental barrier. The basal plate is the base of the intervillous space and is part of the connection between the mother and the fetus. In humans and rats, it shows a good deal of similarity in terms of anatomical location, but not necessarily in terms of morphology. Therefore, more attention should be paid when studying toxicological effects in the basal zone and comparing rat and human placentas. The decidua, the part of the uterine wall that fuses with the chorion, contains many natural killer cells, which are also known in humans as large granular lymphocytes. Many of them cluster around the spiral arteries and play a role in the control of trophoblast invasion and vascular remodeling. These cells thus have approximately the same function in humans as they do in rats [34].

In Europe, babesiosis is perceived mainly as an animal disease, however, awareness that *Babesia* spp. parasites can cause diseases in humans is increasing significantly. Ticks, which are the main vector of these parasites, inhabit areas of human activity: not only forests, as was commonly believed, but also city parks and recreational areas. Recent reports indicate an increasing number of ticks capable of transmitting *Babesia* spp. Therefore, babesiosis has been classified as an emerging infectious disease [1,35].

Cases of congenital invasion by parasites of the genus *Babesia* are becoming increasingly frequent. Vertical transmission is the least-known and least-understood way of spreading the parasite. The vertical route of transmission of *Babesia* spp. has been observed in the following mammals: cattle (*B. divergens*), sheep (*B. ovis*), dogs (*B. gibsoni*), and horses (*B. caballi*, *B. equi*) [36]. There are also reports of congenital *B. microti* infections in humans [37]. Histopathological examination of the placentas of infected females may allow for a better understanding of babesiosis in newborns, and observations of changes in the structure of the placenta will allow us to discover the causes of symptoms occurring in the offspring. 

In this experiment, the presence of *B. microti* in the erythrocytes was observed in the microscopic image, confirming the vertical transmission of the parasites. Studies conducted by Bednarska et al. on BALB/c mice also confirm the ability of *B. microti* to transmit vertically. In addition, the research also suggests that the ability to invade the offspring of an infected female may depend on the phase/intensity of the parasitic infection [36].

Studies were also conducted that focused on histopathological and hematological changes in rat tissues. It was observed that the structure of the liver, kidney, and lung of the infected rat changed due to the congestion of these organs. In addition, in regions of the most severe parasitemia, organs showed swelling and dilation of vessels. In the sinus-type capillaries (sinusoids) in the liver, macrophages and erythrocytes containing parasites were found [38,39,40]. Kupffer cells were enlarged and showed features of phagocytic cells. There was also severe inflammation in the liver, characterized by lymphocyte infiltration [41]. 

In this study, features of organ hyperemia and an increase in perivascular space were also observed. In addition, the presence of lymphocytes in the microscopic image may confirm the presence of ongoing inflammation. The presence of vacuoles in the trophoblast and syncytiotrophoblast may be a consequence of significant disturbances in lipid metabolism and fat metabolism, both of which are observed during pathological processes. Cell vacuolation has also been observed in kidney and liver cells in other studies [42,43]. 

Changes in the spleens of infected rats have also been noted. Imaging of blood cells showed infected erythrocytes, which were characterized by an irregular surface. A more detailed analysis revealed characteristic elongated structures (about 2 µm long) under the cell membrane. The shape of these structures resembled that of *B. microti* merozoites [44]. In the analyzed preparations, erythrocytes containing *B. microti* trophozoites were observed in splenic vessels. In addition, broad bands of fibrin, much larger than those in control animals, were also observed, as in the studies performed on spleens. Fragments of damaged cells were located in intercellular spaces. Many thrombocytes were visible in their vicinity. Disintegrating erythrocytes could be seen in many fields of view. Many damaged cells were also observed in the area of blood vessels, and the cytoplasm of these cells contained a large amount of glycogen and residual bodies. The presence of platelets adhering to the surface of damaged cells, near the vacuole, was also observed [45,46]. 

Tests carried out on placentas revealed similar changes. The pathological features visible in the microscopic image of the examined placentas included the presence of numerous vacuoles within the cells. In the glycogen cells, as in the animals from the control group, the cytoplasm was highly spread, frothy, and devoid of storage materials. The microscopic image of the examined placentas also indicated significant hyperemia of this organ. In some sections, the characteristic adhesion of blood cells (erythrocytes and thrombocytes) to the vascular endothelial cells was visible. Clots were visible in some cross-sections of the vessels. An increased amount of collagen fibers and an enlarged perivascular space were also observed around the blood vessels.

Studies have also been conducted on the livers of rats infected with *B. microti*. During the morphological analysis of the liver structure, numerous lymphocytic infiltrates were observed using light microscopy. The increase in the number of lymphocytes compared to the control group suggested an inflammatory basis of liver damage in the course of babesiosis. In chronic babesiosis, significant damage to hepatocytes was also observed compared to the control. The cells were overgrown, and the cytoplasm was vacuolated. Many cells in the cleavage phase were observed in the blood vessels. Ultrastructural analysis of the perivascular space showed the presence of cellular debris in the vicinity of the vessels [47,48,49].

This study found numerous structural changes in the placenta, as well as abnormalities in the morphology of red blood cells, such as poikilocytosis, anisocytosis, hyperpigmentation, and hypochromia. Previous studies have shown changes in the structure of the villi, including the accumulation of collagen fibers and the expansion of the space around the villi. A characteristic feature of the placentas of females infected with babesiosis is a greater amount of fibrin precipitated in the maternal and fetal blood vessels than is seen in healthy animals. 

From a public-health point of view, babesiosis seems to be of increasing importance [50]. There has been an increase in the number of asymptomatic carriers of *Babesia* spp. The asymptomatic phase of babesiosis can persist for months or even years. Many parasite-infected patients never experience clinical symptoms [51]. The study presents observations concerning changes in the histopathological structure of the placenta in infected female rats. These studies confirm the vertical transmission of *B. microti*. These protozoa cause changes to the placenta and thus can cause placental dysfunction. Newborns are at risk of severe babesiosis and may suffer from some of the complications usually seen in older patients, including respiratory distress syndrome, liver dysfunction, and severe anemia requiring blood transfusion. Obstetricians should be aware of placental babesiosis. Although it is a relatively rare disease, it can be highly virulent and can also be confused with other infections, especially malaria [52]. Each case of human babesiosis should lead to an assessment of the likely route of transmission. In the case of congenital disease, maternal diagnosis is also necessary, as transplacental transmission is likely to be prevented if the maternal condition is diagnosed and treated. As the incidence and diagnosis of babesiosis continues to increase, new reports of congenital babesiosis are expected and should contribute to a better understanding of this rare condition [53,54,55].

## 5. Conclusions

Studies conducted using light microscopy confirmed the vertical transmission of *B. microti* protozoa. The presence of piroplasms was observed in both maternal and fetal vessels. *Babesia microti* infection can pose a serious threat to the fetus. It is necessary to make doctors and patients aware of the existing risk of babesiosis and its complications. Understanding the mechanism of babesiosis transmission still remains a serious research problem.

## Data Availability

Data supporting reported results are available by contacting the corresponding author.
